# Functional Peptides Encoded by Long Non-Coding RNAs in Gastrointestinal Cancer

**DOI:** 10.3389/fonc.2021.777374

**Published:** 2021-11-23

**Authors:** Yao Chen, Weili Long, Liqiong Yang, Yueshui Zhao, Xu Wu, Mingxing Li, Fukuan Du, Yu Chen, Zhihui Yang, Qinglian Wen, Tao Yi, Zhangang Xiao, Jing Shen

**Affiliations:** ^1^ Laboratory of Molecular Pharmacology, Department of Pharmacology, School of Pharmacy, Southwest Medical University, Luzhou, China; ^2^ South Sichuan Institute of Translational Medicine, Luzhou, China; ^3^ Laboratory of Personalised Cell Therapy & Cell Medicines, School of Pharmacy, Southwest Medical University, Luzhou, China; ^4^ School of Basic Medicine, Southwest Medical University, Luzhou, China; ^5^ Department of Pathology, The Affiliated Hospital of Southwest Medical University, Luzhou, China; ^6^ Department of Oncology, The Affiliated Hospital of Southwest Medical University, Luzhou, China; ^7^ School of Chinese Medicine, Hong Kong Baptist University, Hong Kong, Hong Kong SAR, China

**Keywords:** long non-coding RNA (lncRNA), ORF, small peptide, gastrointestinal cancer, function

## Abstract

Gastrointestinal cancer is by far the most common malignancy and the most common cause of cancer-related deaths worldwide. Recent studies have shown that long non-coding RNAs (lncRNAs) play an important role in the epigenetic regulation of cancer cells and regulate tumor progression by affecting chromatin modifications, gene transcription, translation, and sponge to miRNAs. In particular, lncRNA has recently been found to possess open reading frame (ORF), which can encode functional small peptides or proteins. These peptides interact with its targets to regulate transcription or the signal axis, thus promoting or inhibiting the occurrence and development of tumors. In this review, we summarize the involvement of lncRNAs and the function of lncRNAs encoded small peptides in gastrointestinal cancer.

## 1 Introduction

From data obtained during the ENCODE project, it is estimated that the human genome contains both protein-coding and non-coding regions, with less than 2% annotated as protein-coding (1). Most of the non-protein-coding parts of the human genome have long been considered as junk DNA. With the development of deep sequencing technology, a large number of previously unknown transcripts have been identified. The vast majority of these transcripts (>99%) are thought to be long non-coding RNAs (lncRNAs) due to the lack of obvious long protein-coding open reading frame (ORF) and clear homologs in other organisms (2). LncRNAs are a class of important non-coding RNAs, whose transcripts are longer than 200 nucleotides (nt), and have no obvious protein-coding function and no sequence similarity. LncRNAs can be further classified as antisense, intronic, intergenic, and enhancer lncRNAs (3). LncRNAs can perform numerous molecular functions, including regulating transcription patterns, regulating protein activity, performing structural or organizational functions, altering RNA processing events, and acting as precursors for small RNAs (4). In cancer, many lncRNAs are dysregulated, and some of them has been proved to be very specific and sensitive tumor markers, like PCA3 in prostate tumors (5, 6). In previous studies, due to the non-coding nature of lncRNAs, a large number of lncRNAs were thought to function in cancer through their own transcripts. However, recent studies have revealed that lncRNA can encode small peptides through transcripts which can bind ribosomes to performs biological functions, which is a new breakthrough in the study of lncRNA (7).

Gastrointestinal cancers comprise the esophagus, stomach, liver, bile duct, gallbladder, pancreas, colon, appendix or rectum cancer, which together constitute the leading cause of cancer-related deaths worldwide (8). Obesity, smoking, Helicobacter Pylori, hepatitis C virus (HCV) and hepatitis B virus (HBV) are known environmental risk factors for the development of gastrointestinal cancer (9). Despite the advances of chemotherapy in the treatment of some gastrointestinal cancers, the prognosis for many patients remains poor, with a 5-year survival rate of less than 10% (10, 11). Current treatment strategies also include radiotherapy, surgery and targeted therapy. Immunotherapy like checkpoint inhibitors blocking PD-1 and cytotoxic T lymphocyte associated antigen 4(CTLA-4) immunotherapy may benefit patients, but further clinical trials are needed (12).

Given the paucity of treatment options for gastrointestinal cancers, new therapeutic target is urgently needed. The clinical implication of the bifunctional lncRNAs awaits further exploration. In this review, we provide a brief overview of the role of lncRNA and especially summarize the function of small peptides encoded by lncRNAs in gastrointestinal cancers.

## 2 Function of lncRNAs in Gastrointestinal Cancer

**Figure 1:**
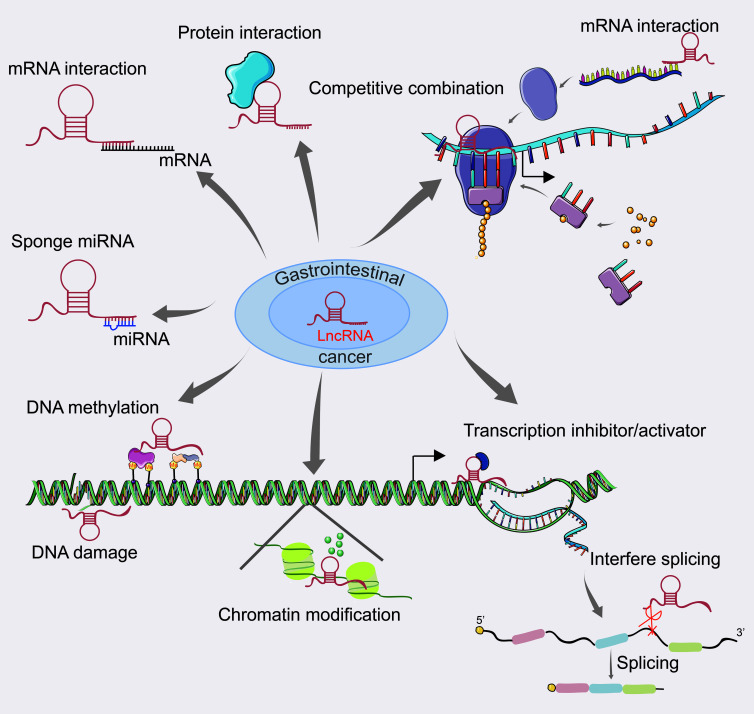
The role of lncRNA in gastrointestinal cancer.

According to global cancer statistics, cancer of the digestive tract is the leading cause of cancer and cancer-related deaths (8). Recent studies have shown that abnormal lncRNA expression is related to tumorigenesis, progression, invasion, and overall survival in patients with gastrointestinal cancer (13). Studies of lncRNA dysregulation in gastrointestinal cancer are shown in **Supplementary Table 1**. LncRNAs have been identified as important players in gene regulation. LncRNA regulates gene expression mainly by participating in transcriptional regulation, such as inhibited RNA polymerase II, chromatin remodeling and histone modification, interfered mRNA splicing, and complementary double chains formed with the transcripts of the protein coding genes and endogenous siRNA produced in response to the Dicer enzyme to interfere the expression (4). In addition, LncRNAs can affect post-transcriptional processing, act as a structural component to form nucleic acid protein complex with the protein, regulate the activity of the protein or change the cellular localization of the protein and even as a “sponge small molecule competitive effect on the role of endogenous RNA, etc. (14, 15). The role of lncRNAs in gastrointestinal cancer is shown in **Figure 1**.

### 2.1 Transcriptional Regulation

#### 2.1.1 DNA Methylation and DNA Repair

DNA methylation occurs in gene promoters, genomes, and intergenic regions of the genome, and plays a key role in both chromatin organization and gene expression (16). Differential genes affected by lncRNA lincDUSP were mainly enriched in DNA damage response and cell cycle control pathways. In addition, the identification of the locus of lincDUSP chromatin by ChIRP-Seq showed that it was associated with genes involved in replication-related DNA damage responses and cell cycle control. Further studies showed that deletion of the lincDUSP gene in colon cancer cell lines increased the accumulation of early s-phase cells and the formation of γH2AX lesions, suggesting increased induction of DNA damage response (17). LncRNA DACOR1 induces down regulation of cystathionine synthetase, resulting in elevated levels of s-adenosylmethionine, a key methyl donor for DNA methylation, suggesting that DACOR1 leads to abnormal DNA methylation and the occurrence of colon cancer (18). LncRNA HOTAIR may enhance 5-fluorouracil-induced apoptosis by reducing the methylation of the MTHFR promoter in esophageal cancer cells (19).

#### 2.1.2 Chromatin Modification

Some lncRNAs can also bind to nucleosome mobilization complexes to mediate chromatin structure remodeling, thus modulating gene expression. Most lncRNAs work with DNA-binding proteins, such as chromatin-modifying complexes, and regulate the expression of multiple genes through epigenetic regulation (20–22). The expression of lncRNA HOTAIR is associated with PRC2 -functional genome-wide reprogramming not only in breast cancer but also in colorectal cancer. It regulates polycomb-dependent chromatin modification, and its upregulation may be a key factor in metastasis progression (23). LncRNA NMR is a novel NSUN2 methylated lncRNA, upregulated in esophageal squamous cell carcinoma (ESCC), functioned as a key factor of ESCC progress. NMR can directly bind to BPTF, which is a chromatin regulator that contributes to ATP-dependent chromatin remodeling (24), and promote the expression of MMP3 and MMP10 by ERK1/2 pathway through recruiting BPTF to chromatin (25).

#### 2.1.3 Alternative Splicing

Alternative splicing (AS) can selectively remove introns, retain exons, and produce two or more mRNA splicing isomers, thus increasing the diversity of gene phenotypes and protein functions in eukaryotes (26, 27). In recent years, many AS changes have been found to play an important role in the development of cancer. With the development of high-throughput sequencing and RNA-seq techniques, thousands of non-coding RNAs have been identified as critical for regulating AS at multiple levels in cancer (28). LncRNA DGCR5 binds directly to serine-and arginine-rich splicing factor 1(SRSF1) to increase its stability, thereby stimulating the Mcl-1 alternative splicing event to promoting the occurrence of esophageal squamous-cell carcinoma *in vivo* (29). LncRNA MALAT1 promotes cell proliferation, migration and invasion by increasing AKAP-9 expression in colorectal cancer cells through SRPK1-catalyzed phosphorylation of SRSF1 (30). Furthermore, SRPK1 phosphorylation of SRSF1 was found to modulate the alternative splicing of Rac1b in colorectal cells (31).

#### 2.1.4 Transcription

Transcription is the transfer of genetic information from the DNA level to the RNA level, it is the key step for genes to perform their functions. Currently, lncRNA has been found to be involved in regulating gene transcription (32). Furthermore, lncRNA has also been found to be involved in transcriptional regulation that affects the development of gastrointestinal cancer. In colorectal cancer, lncRNA HITT, in cooperation with Ezh2, inhibits HIF-1α transcription and inhibits hypoxia adaptation in colorectal cancer cells (33). Besides, lncRNA SATB2-AS1 directly binds to WDR5 and GADD45A, cis-activating SATB2 transcription *via* mediating histone H3 lysine 4 tri-methylation deposition and DNA demethylation of the promoter region of SATB2, thus inhibiting CRC transfer and modulating CRC immune response (34). In addition, lncRNA has also been found to regulate cancer by silencing gene expression. AS1DHRS4 is a natural antisense head to head transcript, which can silence the DHRS4 gene cluster in cis and trans form (35). LncRNA CASC2 silences BCL2 expression through EZH2-dependent binding to the promoter region of BCL2, thus enhancing the cytotoxicity of berberine-induced colorectal cancer cells (36). Similarly, lncRNA HOXA transcript at the distal tip promotes colorectal cancer growth partially *via* silencing of p21 expression (37).

### 2.2 Translational Regulation

The control of translation is often considered a major factor in determining protein levels in cells. LncRNAs can affect tumor progression by regulating the translation of coding RNAs. LncRNA GMAN, overexpressed in gastric cancer, regulates ephrin A1 mRNA translation through competitive binding with GMAN-As, thus promoting proliferation and metastasis in gastric cancer (38). In addition, lncRNA can also act by binding to ribosomal mRNAs. Ribosomes, organelles that mediate protein synthesis, are often typically seen as a key factor in the translation process and ribosome recruitment is seen as a regulatory endpoint (39). Till now, there are few studies on the mechanism of lncRNA affecting the translation process through the interaction with ribosomal proteins in gastrointestinal cancer. In colorectal cancer, lncRNA LUCAT1 binds to UBA52, which encodes ubiquitin and 60s ribosomal protein L40(RPL40), and affects its stability in a proteasome dependent manner (40). Equally, ribosomal protein S24 mRNA binds to lncRNA MVIH to enhance their stability, thereby activating angiogenesis in colorectal cancer by inhibiting the secretion of PGK1 (41).

### 2.3 miRNA Sponge

In addition to binding to coding RNA and proteins, lncRNAs have also been found to act as sponge by binding to miRNA molecules and reducing their ability to target mRNAs in gastrointestinal cancers. In colorectal cancer, lncRNA MALAT1 can sponge miR-106b-5p and enhanced microtubule migration *via* SLAIN2 to promote invasion and metastasis (42). LncRNA 00152 acts as competitive endogenous RNA and modulates NRP1 expression by sponging with miRNA-206, thus promoting cancer progression in colorectal cancer (43). In gastric cancer, lncRNA LINC01133 is down-regulated in gastric cancer tissues and cell lines, and acts as competitive endogenous RNA (ceRNA) *via* sponging miR-106a-3p to regulate APC expression and Wnt/β-catenin pathway to inhibit gastric cancer progression and metastasis (44). Similarly, lncRNA XIST acts as ceRNA to inhibit the miR-497/MACC1 axis and promote the growth and invasion of gastric cancer cells (45). In addition, there are also studies on the biological function of lncRNAs through “sponging” miRNA in liver cancer (46), esophageal cancer (47), pancreatic cancer (48), cholangiocarcinoma (49) and gallbladder cancer (50), indicating that lncRNAs’ role in “sponging” miRNAs has significant significance in gastrointestinal cancer.

## 3 LncRNA Encoded Small Peptides

Although lncRNAs are defined as transcripts without coding ability, many studies in recent years have found open reading frame (ORF) in lncRNAs by bioinformatics analysis (51). Recent studies have also found that some lncRNAs appear to be highly correlated with the ribosome, suggesting that lncRNA may contain coding regions for translation that can encode short-chain small peptides (52). Analysis of ribosomal map data showed that 40% of lncRNAs and pseudogene RNA expressed in human cells were translated, and mass spectrometry confirmed that lncRNAs could indeed be translated into small peptides (53, 54). Sebastian et al. analyzed 354 translatable open reading frames from lncRNAs and obtained 22 small peptides from mass spectrometry (55). The translated lncRNAs are preferentially located in the cytoplasm, while the untranslated lncRNAs are preferentially located in the nucleus. The translation efficiency of cytoplasmic lncRNAs is almost equal to mRNAs, indicating that cytoplasmic lncRNAs are bound by ribosomes and translated, but most of the small peptides produced by lncRNAs may be highly unstable by-products and have no function (53).

Although many small peptides encoded by lncRNA ORF have been found so far, only part of their biological functions have been revealed. These small peptides are usually conserved and involved in a wide range of biological processes (7). LncRNA-encoded small peptides play important roles in inflammation, signal transduction and metabolism *etc.* (56, 57). LncRNA lncVLDLR can encode a 44-aa inflammation-modulating micropeptide (IMP) that can participate in inflammatory responses by interacting with transcriptional coactivators (56). LncRNA linc00116 encodes a 56-aa peptide named Mtln that can connect respiration and lipid metabolism (58). Micropeptide encoded by lncRNA Toddler can accelerate gastrula formation by activating APJ/Apelin receptor signals (59). Recent studies have also found that these small peptides encoded by lncRNAs can play a vital role in the development of cancer (**Table 1**).

**Table 1:** Tumor-related functional peptides encoding by lncRNAs.

LncRNA	Peptide name	Peptide length	Cancer type	Functions of peptides	References
lncRNA HOXB-AS3	HOXB-AS3	53 aa	Colon Cancer	Suppress colon cancer aerobic glycolysis by blocking hnRNP A1 dependent PKM splicing and PKM2 formation.	(60)
lncRNA HOXB-AS3	–	–	Oral squamous cell carcinoma	Bind with IGF2BP2 to stabilize c-Myc, thus promoting the cancer cell proliferation and viability.	(61)
LINC01420	NoBody	71 aa	Lung cancer	Participate in mRNA processing, nonsense-mediated decay (NMD) and negatively regulate P-body association.	(62)
LncRNA UBAP1-AST6	UBAP1-AST6	12.8 kDa	Colon cancer Lung cancer	Act as a possible tumor promoter and can significantly promote cell proliferation and clone formation.	(63)
LINC00908	ASRPS	60 aa	Triple-negative breast cancer	Bind to STAT3 and down-regulates STAT3 phosphorylation, resulting in decreased VEGF expression.	(64)
LINC00665	CIP2A-BP	52 aa	Triple-negative breast cancer	Inhibit triple negative breast cancer by PI3K/AKT/NF κB pathway.	(65)
lncRNA CASIMO1	SMIM22	10 kDa	Breast cancer	Control cell proliferation and interacts with squalene oxidase to regulate lipid droplet formation.	(66)
lncRNA meloe	MELOE-3	54 aa	melanoma	The polypeptide MELOE-3 has immune tolerance.	(67)
LncRNA CRNDE	CRNDEP	84 aa	Hela cell	May be involved in the regulation of cell proliferation and oxygen metabolism	(68)
LINC00675	FORCP	79 aa	colorectal cancer	Inhibit proliferation, colon formation and tumorigenesis.	(69)
LOC90024	SRSP	130 aa	colorectal cancer	Interact with RNA splicing regulator SRSF3 to regulate mRNA splicing, thus inducing “carcinogenesis”.	(70)
LINC00266-1	RBRP	71 aa	colorectal cancer	Interact with RNA-binding proteins, including the m6A reader IGF2BP1, to induce cancer.	(71)
LINC00467	ASAP	94 aa	Colorectal cancer	Interact with subunits α and γ (ATP5A and ATP5C) to enhance the construction of ATP synthase, increase the activity of ATP synthase and the rate of mitochondrial oxygen consumption, thus promoting the proliferation of colorectal cancer cells.	(72)
LINC00998	SMIM30	59 aa	Liver cancer	Promote the proliferation, migration and invasion of liver cancer.	(73)
lncRNA HBVPTPAP	HBVPTPAP	145 aa	Liver cancer	Interaction with PILRA intracellular domain to activate downstream JAK/STAT signaling pathway to induce apoptosis of liver cancer cells.	(74)
lncRNA NCBP2-AS2	KRASIM	99 aa	Liver cancer	Interact with KRAS protein to inhibit ERK signaling, leading to the suppression of liver cancer cell growth.	(75)
lncRNA ZFAS1	ZFAS1	–	Liver cancer	Down-regulate NADH dehydrogenase expression (NDUFA6, NDUFB4, and NDUFB11) to enhance ROS production and thus promote cancer cell migration.	(76)
LINC-PINT	PINT87aa	87 aa	Liver cancer	Induce cell cycle arrest and cell Senescence by directly binding to FOXM1 to block PHB2 transcription	(77)
LncRNA RP11-469H8.6	MIAC	51 aa	Head and neck squamous cell carcinoma (HNSCC)	Interact with AQP2 to inhibit tumor growth and metastasis of HNSCC by regulating the actin cytoskeleton of SEPT2 and ITGB4.	(78)
LINC00278	YY1BM	21 aa	Esophageal squamous cancer	Inhibit the interaction between YY1 and androgen receptor (AR), thus reducing the expression of eEF2K through AR signaling pathway.	(79)
DLEU1	ORF1 and ORF8 small peptide	–	Glioma	Act as a self-assembly water channel leads to increased permeability of Glioma, which leads to brain edema and even increases the risk of invasion and metastasis of cancer cells.	(80)

**Figure 2:**
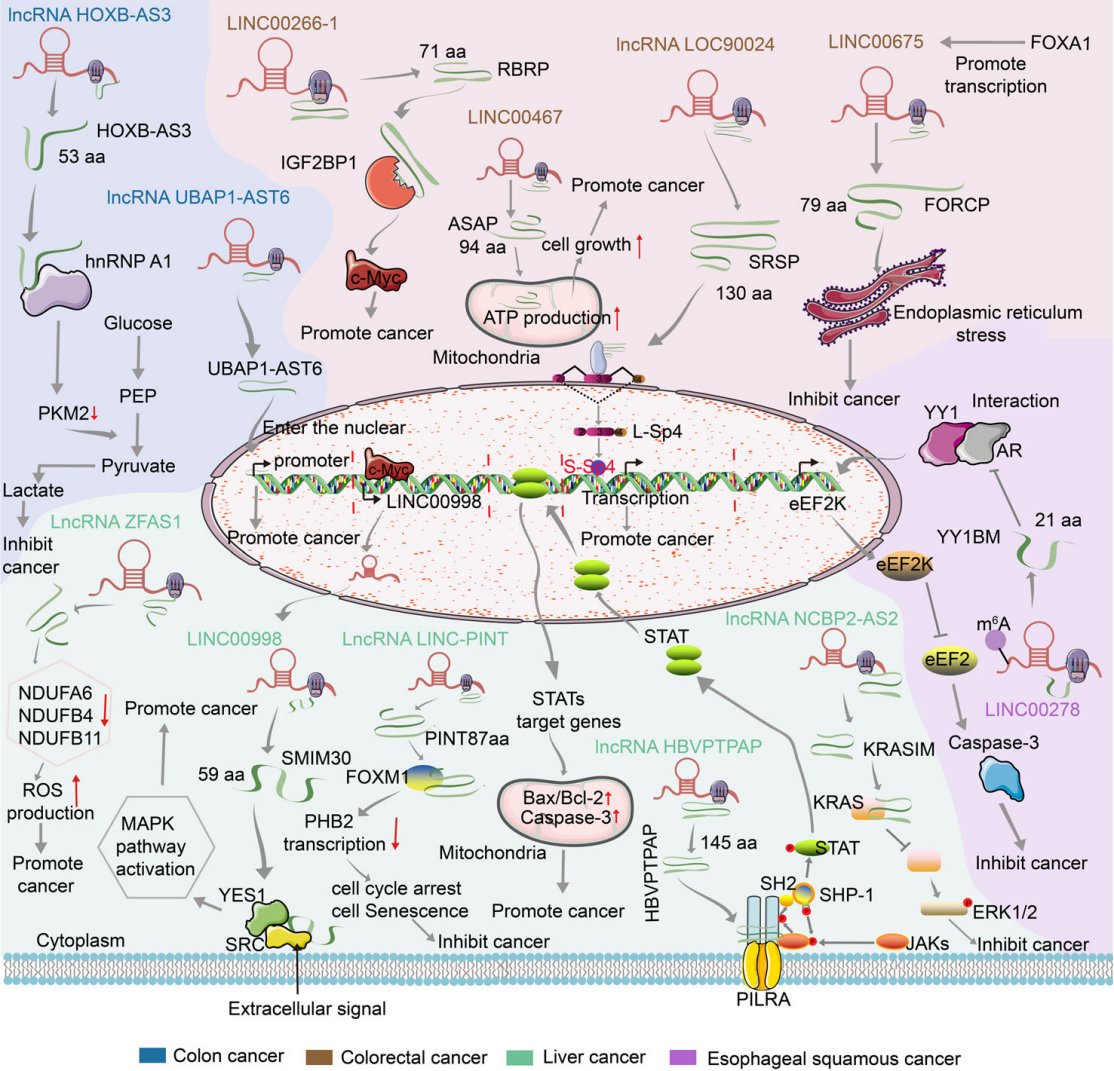
Mechanism of action of lncRNA encoded small peptides in gastrointestinal cancer.

At present, several functional peptides encoded by lncRNA have been found in gastrointestinal cancer. Their RNA level studies are shown in **Table 2** and their mechanism of action by encoding small peptides is summarized in **Figure 2**.

**Table 2:** Studies of lncRNAs at RNA level.

lncRNA	Cancer type	Mechanism	Functions	References
LncRNA HOXB-AS3	Epithelial ovarian cancer	Sponge miR-378a-3p.	Change glycolysis to promote growth, invasion and migration.	(81)
	Lung cancer	Activate PI3K-AKTpathway.	Promote proliferation, migration and invasion.	(82)
	Liver cancer	Inhibit P53 expression by combination with DNMT1.	Promote cell proliferation, inhibit cell apoptosis and induce cell cycle arrest in G0/G1 phase.	(83)
	Acute myeloid leukemia	Interact with ErbB3-binding protein 1(EBP1) and directs EBP1 to the ribosomal DNA site.	Promote cancer progression	(84)
	Acute myeloid leukemia	Enhance cell cycle processes and DNA replication.	Promotes bone marrow cell proliferation	(85)
	Endometrial Cancer	Target miR-498-5p to regulate the expression of ADAM9.	Promote cell proliferation and inhibit cell apoptosis.	(86)
	Epithelial ovarian cancer	Enhance Wnt/β-catenin signal activity.	Promote cell invasion, migration and proliferation.	(87)
LINC00675	Clear cell renal cell carcinoma	Activate Wnt/β-catenin pathway.	Inhibit proliferation, migration, and invasion.	(88)
	Prostate cancer	Activate LINC00675/MDM2/GATA2/AR signaling axis.	Promote castration-resistant prostate cancer progression	(89)
	Gastric cancer	Down-regulate the H3K4me2 level of SPRY4 promoter.	Inhibits cell proliferation and migration.	(90)
	Glioma	Up-regulate of TRIP6 expression	Promote cell proliferation, migration and invasion.	(91)
	Bladder cancer	Suppress β-catenin and its downstream gene expression	Inhibit migration, invasion and proliferation.	(92)
	Liver cancer	Modulate miR-942-5p and GFI1.	Inhibiting metastasis.	(93)
	Cervical cancer	Up-regulate Wnt/β-catenin signal activity.	Promote proliferation, migration and invasion of cancer cells, and inhibit cell apoptosis.	(94)
	Esophageal squamous-cell carcinoma	Inhibit Wnt/β-catenin signal transduction.	Inhibit tumorigenesis and the epithelial-mesenchymal transition.	(95)
	Colorectal cancer	Down-regulate miR-942 and suppress WNT/-Catenin signal.	Inhibit cell proliferation and metastasis.	(96)
	Ductal adenocarcinoma of pancreas	–	Promote proliferation and invasion.	(97)
	Pancreatic cancer	–	New biomarkers of pancreatic cancer.	(98)
	Gastric cancer	Interact with vimentin and enhance its phosphorylation on Ser83.	Inhibit proliferation, migration and invasion, and inhibit the distal lung and liver metastasis *in vivo.*	(99)
	Oral squamous-cell carcinoma	Acts on miR-103a-3p.	Inhibit proliferation and induce apoptosis.	(100)
	Colorectal Cancer	Copy number variation.	Leads to poor prognosis.	(101)
LINC00266-1	Osteosarcoma	Activate LINC00266-1/miR-548c-3p/SMAD2 feedback loop.	Promote the development of osteosarcoma.	(102)
LINC00998	Malignant glioma	Modulate the axis of c-Met/AKT/mTOR mediated by CBX3.	Inhibit the progression of glioma.	(103)
lncRNA NCBP2-AS2	Non-small cell lung cancer	May modulate cancer-related Wnt signaling pathway.	Related to the occurrence and development of lung cancer.	(104)
LINC00467	Colorectal cancer	cyclin D1, cyclin A1, CDK2, CDK4 and Twist1 ↓ E-cadherin ↑	Promote the occurrence and development of colorectal cancer.	(105)
	Colorectal cancer	Target miR-451a.	Promote cell proliferation and metastasis and inhibit apoptosis.	(106)
	Colorectal cancer	Competed with Ferritin Light Chain (FTL) for mir-133b binding sites.	Regulate metastasis and chemical resistance.	(107)
lncRNA ZFAS1	Liver cancer	–	Be used as a biomarker of liver cancer.	(108, 109)
	Liver cancer	Inhibit miR-193a-3p.	Enhance the proliferation of liver cancer cells.	(110)
	Liver cancer	PERK/ATF4/ZFAS1 signaling axis.	Associate with Sorafenib Resistance in liver cancer.	(111)

↑: Up-regulated expression; ↓: Down-regulated expression.

### 3.1 LncRNA HOXB-AS3

LncRNA HOXB-AS3 was found to be associated with tumorigenesis and development since its discovery. First reported by J Huang and colleagues, lncRNA HOXB-AS3 can encode a conserved 53-aa peptide to inhibit the growth of colon cancer (60). In the following studies, although the effects of lncRNA HOXB-AS3 on other tumors were also reported, it is interesting to note that the studies were only at the RNA level and mostly showed cancer-promoting properties. For example, low expression of HOXB-AS3 can reduce the expression of lactate dehydrogenase A and extracellular acidification rate through the sponge miR-378a-3p, thus promoting the growth, invasion and migration of epithelial ovarian cancer (81). The expression of HOXB-AS3 was significantly increased in non-small-cell lung carcinoma tissues and cells, and the activation of PI3K-AKT increased the proliferation, migration and invasion of lung cancer (82). In acute myeloid leukemia, HOXB-AS3 is a poor prognostic marker that interacts with ErbB3-binding protein 1 and leads ErbB3-binding protein 1 to ribosomal DNA sites to regulate ribosomal RNA transcription and protein synthesis from scratch to promote tumor (84, 85). HOXB-AS3 can also promote the tumorigenesis of epithelial ovarian cancer through Wnt/β-catenin signaling pathway (87), and promote endometrial cancer cell proliferation and inhibit apoptosis by targeting miR-498-5p to regulate ADAM9 expression (86). In addition to its effect on tumors, HOXB-AS3 has been reported to inhibit cardiomyocyte proliferation induced by targeting and down regulation of miRNA-875-3p protection (112).

In gastrointestinal cancers, studies of HOXB-AS3 at RNA levels have shown a promoting effect on liver cancer. HOXB-AS3 is highly expressed in liver cancer tissues, and can inhibit cell apoptosis, promote proliferation and induce cell cycle stagnation in G0/G1 phase by binding with DNMT1 locus to inhibit the expression of p53 (83). In colon cancer, HOXB-AS3 can competitively bind to arginine residues in the RGG motif of hnRNP A1 by encoding a conserved 53-aa peptide, thus blocking hnRNP A1 dependent pyruvate kinase M(PKM) splicing, PKM2 formation and subsequent colon cancer cell metabolic reprogramming to inhibit tumorigenesis. The mean overall survival time of colon cancer patients with high HOXB-AS3 peptide expression was 1.6 times that of those with low HOXB-AS3 peptide expression, suggesting that high HOXB-AS3 peptide expression may reduce the risk of death. Further studies showed that HOXB-AS3 peptide, not lncRNA HOXB-AS3, inhibits colon cancer cell growth, invasion, migration, colony formation, and tumor growth *in vitro* and *in vivo* (60).

### 3.2 LncRNA UBAP1-AST6

Sequencing of full-length translated mRNA by different studies from human colorectal cancer (113), liver cancer (114), and cervical cancer (115) showed that 1028 ~ 3330 lncRNAs were bound to ribosomes. Among them, 2969 lncRNAs had canonical open reading frames (ORFs) beginning with AUG, which could encode proteins of at least 50 amino acids in length. Mass spectrometry and western blot analysis by Shaohua L et al. confirmed that lncRNA UBAP1-AST6 could encode a peptide. The function-related translation ratio (TR), defined as the ratio of mRNA to total mRNA of a gene, was found to be significantly higher in the average TR of lncRNA than in known coding genes, indicating that the translation of new proteins was more active. Localization of EGFP and mCherry fusion protein revealed that UBAP1-AST6 was localized in the nucleus. UBAP1-AST6 acts as a possible tumor promoter in its protein form, and its overexpression can significantly promote cell proliferation and clone formation, but not in its UBAP1-AST6 RNA form (63).

### 3.3 LncRNA LINC00675

LINC00675 was found to have both anti-cancer and pro-cancer effects. However, in gastrointestinal carcinoma, LINC00675 mainly shows tumor suppressor properties. Shuo Z et al. found that LINC00675 enhanced the phosphorylation of vimentin on Ser83 to inhibit the progression of gastric cancer (99). LINC00675 inhibits cell proliferation and migration in colorectal cancer by acting on miR-942 and Wnt/β-catenin signaling (96). LINC00675 can also inhibit tumorigenesis and epithelial-mesenchymal transition by inhibiting the Wnt/β-catenin signaling pathway in esophageal squamous cell carcinoma (95).

A recent study shows that LINC00675 is regulated by the precursor transcription factor FOXA1 and can encode a small conserved 79-amino acid protein called FORCP in colorectal cancer. The FORCP protein is mainly located in the endoplasmic reticulum. The FORCP transcript was not detected in most cell types, but was abundant in well-differentiated colorectal cancer cells, suggesting that FORCP is a novel small conserved protein encoded by misannotated lncRNA. FORCP can inhibit cell proliferation, clone formation and tumorigenesis (69).

### 3.4 LncRNA LOC90024

Nan M et al. used RNA sequencing to purify ribosomal-bound RNA and found that lncRNA LOC90024 could bind to the ribosome, suggesting that LOC90024 might be translated into a protein/peptide. LncRNA LOC90024 was confirmed to encode a small protein SRSP of 130 amino acids by GFP fusion protein, mass spectrometry and western blot assay. SRSP is endogenous and produced naturally in human cells and tissues. Increased LOC90024 and SRSP levels are associated with poor prognosis in CRC patients. However, SRSP rather than LOC90024 lncRNA itself promoted CRC tumorigenesis. Mechanistically, SRSP acts on RNA splicing regulator SRSF3 to regulate mRNA splicing. SRSP increased the binding of SRSF3 to exon 3 of transcription factor Sp4, which promoted the formation of “ oncogene” long Sp4 subtype (L-Sp4 protein) and inhibited the formation of “tumor suppressor” short Sp4 isoform (S-Sp4 peptide). Overall, the results reveal that small lncRNA-encoded protein SRSP induces “carcinogenesis” (70).

### 3.5 LncRNA LINC00266-1

In RNA level studies, LINC00266-1 has been found to promote the development of osteosarcoma by stimulating the LINC00266-1/mir-548c-3p/SMAD2 feedback loop (102). Song Z et al. found that LINC00266 could encode a 71 aa polypeptide, which was named “RBRP” as it mainly interacts with RNA-binding proteins, including m6A reader IGF2BP1. RBRP is expressed naturally and internally. Compared with the control group, RBRP was up-regulated in CRC tissues and cells, and the high expression of RBRP was an independent prognostic factor in CRC patients. LINC00266-1 lncRNA and RBRP expression levels were increased in primary cancer cells and highly metastatic cancer cells, but RBRP peptide, not lncRNA LinC00266-1 itself, promoted tumorigenesis. RBRP enhances mRNA stability and expression of c-Myc by binding to IGF2BP1 and enhancing m6A recognition by IGF2BP1 on RNAs, like c-Myc mRNA, thus promoting tumorigenesis. RBRP is a regulatory subunit of m6A reader and enhances m6A recognition of target RNA by m6A reader to play its carcinogenic role (71).

### 3.6 LINC00467

In colorectal cancer, up-regulation of LINC00467 reduces the expression of cyclin A1, cyclin D1, CDK2, CDK4 and Twist1, and enhances the expression of E-cadherin, thus promoting the development of colorectal cancer (105). Increased expression of LINC00467 promotes cell proliferation and metastasis and inhibits apoptosis by targeting miR-451a (106). Li et al. found that LINC00467 regulates metastasis and chemical resistance of colorectal cancer by competing with Ferritin Light Chain (FTL) for the miR-133b binding site (107). Recent studies have found that lncRNA LINC00467 can encode a 94-aa micropeptide, which is located in mitochondria, to enhance the construction of ATP synthase by interacting with subunits α and γ (ATP5A and ATP5C), thus increasing ATP synthase activity and mitochondrial oxygen consumption rate to promote colorectal cancer cell proliferation (72).

### 3.7 LncRNA LINC00998

Through the RIP-seq analysis of Ribosomal protein S6, Yanan P et al. found that LINC00998 has the ability to encode a 59aa endogenous small peptide, named SMIM30. SMIM30 is a conserved and hydrophobic membrane peptide and highly expressed in HCC. High level of SMIM30 is predictive of poor prognosis. The SMIM30 Peptide, rather than LINC00998, promotes proliferation, invasion and migration of hepatoma cells *in vitro* and *in vivo*. In addition, SMIM30 is transcribed by c-Myc and interacts with non-receptor tyrosine kinases SRC/YES1 to drive membrane anchoring, thus activating MAPK signaling pathways (73).

### 3.8 LncRNA HBVPTPAP

LCRNA HBVPTPAP, which was classified as long non-coding RNA according to the structural characteristics of the full-length sequence, is a new gene screened from HepG2 cell line by Yongzhi L et al. using suppression subtractive hybridization. With the discovery of a unique and complete open reading frame and related coding capability validation, lncRNA HBVPTPAP was found to encode a small 145aa peptide. The subcellular localization of lncRNA HBVPTPAP is mainly in the cytoplasm, which may activate the downstream JAK/STAT signaling pathway through the interaction between the encoded peptide and the PILRA intracellular domain to induce apoptosis of hepatoma cells (74).

### 3.9 lncRNA NCBP2-AS2

In RNA level studies, lncRNA NCBP2-AS2 was found to be abnormally expressed in osteoblasts of osteoporosis patients and may be involved in inflammatory responses (116). Dongbo Zhou et al. found that the differential expression of NCBP2-AS2 is related to the occurrence and development of lung cancer and may regulate cancer-related Wnt signaling pathway (104). In further studies, Wenli Xu et al. found through ribosomal profiling analysis that lncRNA NCBP2-AS2 had the ability to encode a 90-aa small peptide named KRASIM. KRASIM is highly conserved and significantly down-regulated in hepatocellular carcinoma. The high expression of KRASIM can inhibit proliferation, clone formation and cell cycle progression of hepatocellular carcinoma cells. KRASIM interacts with KRAS protein to inhibit ERK signaling, leading to the suppression of hepatocellular carcinoma cell growth (75).

### 3.10 LncRNA ZFAS1

Studies have shown that up-regulation of lncRNA ZFAS1 can promote metastasis in liver cancer (117). LncRNA ZFAS1 has also been reported to be a biomarker for liver cancer (108, 109). H-L Zhou et al. found that lncRNA ZFAS1 enhanced the proliferation of liver cancer by inhibiting miR-193a-3p (110). Recent research evidence has revealed that lncRNA ZFAS1 can encode a small peptide to down-regulate NADH dehydrogenase expression (NDUFA6, NDUFB11 and NDUFB4) to enhance ROS production, thus promoting cancer development (76).

### 3.11 LncRNA LINC-PINT

Xiaohong X et al. used a computer aging model to identify senescence related lncRNAs and detected 6 differentially expressed lncRNAs, in which LINC-PINT was significantly differentially expressed in senescent cells, indicating that it was involved in cellular aging of liver cancer. Subsequent studies found that lncRNA LINC-PINT can encode an 87-aa small peptide named PINT87aa to perform biological functions. PINT87aa can induces cell cycle arrest and cell senescence by directly binding to FOXM1 to block PHB2 transcription (77).

### 3.12 LncRNA LINC00278

By screening differentially expressed lncRNAs in males, Siqi W et al. found that LINC00278 was significantly down-regulated in esophageal squamous cell carcinoma tissues compared with adjacent normal tissues. LINC00278 can encode a 21-amino acid Yin Yang 1 (YY1)-binding micropeptide, named YY1BM. YY1BM inhibits eEF2K transcription by blocking the interaction between YY1 and androgen receptor (AR), thus promoting eEF2 activity and leading to ESCC apoptosis. LINC00278 has a classic m6A modified motif near the termination codon of YY1BM, which can interact with YTHDF1 and in turn promote the translation of YY1BM. However, smoking increased ALKBH5 expression and decreased the m6A modification of LINC00278, thus inhibiting the translation of YY1BM and inducing ESCC progression (79).

## 4 Conclusions and Perspectives

Only about 1.5% of the human genome contains protein-coding genes, and the rest of the genome contains non-coding sequences. Most of these non-coding DNA sequences can be transcribed into RNA, including tens of thousands of lncRNAs and thousands of miRNAs (118). LncRNA has been found to play an important role in gastrointestinal cancer, including through regulating chromatin structure, regulating transcription, translation, and acting as a sponge-miRNA interaction to inhibit or promote cancer (119). But as lncRNA has been known to be non-coding RNA for a long time since it was recognized, these studies are only based on RNA levels. Interestingly, some of these lncRNAs have recently been found to have open reading frame and can regulate the development of tumors by encoding small peptides or proteins. This finding is also reported in gastrointestinal cancers, such as lncRNA HOXB-AS3 and lncRNA UBAP1-AST6 in colon cancer, LINC00675, lncRNA LOC90024, LINC00266-1 and LINC00467 in colorectal cancer, LINC00998, lncRNA HBVPTPAP, lncRNA NCBP2-AS2, lncRNA ZFAS1 and lncRNA LINC-PINT in liver cancer, LINC00278 in asophageal squamous cancer.

Although there have been a large number of studies on lncRNA in gastrointestinal cancer, several aspects are still lacking. First of all, although there are studies on lncRNA regulation of translation, there is still a lack of in-depth research on the regulation of transcription by lncRNA interacting with ribosomal proteins. Second, it is still unclear whether the binding of lncRNA to proteins can affect tumor development by changing protein localization and protein properties in gastrointestinal cancer. Finally, the fact that lncRNA encodes peptides breaks the previous understanding of lncRNA. The mechanisms of lncRNA to perform functions become more complicated. Current studies have shown that the small peptides encoded by lncRNA are involved in the regulation of gastrointestinal cancer. However, more comprehensive and in-depth studies of lncRNA encoded small peptides and their mechanisms are needed before potential application of it in the prevention and treatment of gastrointestinal cancer.

## Author Contributions

YaC and WL searched and reviewed published articles and wrote the manuscript. LY, YZ, XW, ML, FD, YuC, ZY, QW, and TY conducted online data searches and critically reviewed the article. JS and ZX made substantial contributions to the conception, design of the study and made revisions to the manuscript. All authors contributed to the article and approved the submitted version.

## Funding

This work was supported by National Natural Science Foundation of China (No. 81972643, No. 82172962), Sichuan Science and Technology Project (2021YJ0201) and Luxian People’s Government and Southwest Medical University Scientific and Technological Achievements Transfer and Transformation Strategic Cooperation Project (2019LXXNYKD-07).

## Conflict of Interest

The authors declare that the research was conducted in the absence of any commercial or financial relationships that could be construed as a potential conflict of interest.

## Publisher’s Note

All claims expressed in this article are solely those of the authors and do not necessarily represent those of their affiliated organizations, or those of the publisher, the editors and the reviewers. Any product that may be evaluated in this article, or claim that may be made by its manufacturer, is not guaranteed or endorsed by the publisher.
