# Supplementary material for: Functional Peptides Encoded by Long Non-Coding RNAs in Gastrointestinal Cancer
Source: Front Oncol. 2021 Nov 23;11:777374. doi: 10.3389/fonc.2021.777374 (PMC8649637; doi:10.3389/fonc.2021.777374)
Supplement: Supplementary file 1 [file DataSheet_1.pdf]

Supplementary table1. Panel of H5 hemagglutinins used in hemagglutination inhibition assay and pseudotype-based neutralization assay.

Strain	Abbreviation	(Sub)clade	Accession no.	Assay(s)
A/Vietnam/1194/2004	NIBRG-14 (Vaccine strain)	1	ABP51976	HI[1]
A/Vietnam/1203/2004	Vietnam	1	ABP51977	PN[2]
A/Cambodia/R0405050/2007	NIBRG-88	1.1	ACI06178	HI
A/Indonesia/5/2005	Indonesia, IBCDC-RG2	2.1.3.2	ABP51969	HI, PN
A/turkey/Turkey/1/2005	NIBRG-23	2.2.1	ABQ58921	HI
A/Turkey/65596/2006	Turkey	2.2.1	ABQ58925	PN
A/common magpie/Hong Kong/5052/2007	HK5052	2.3.2.1	ACJ26242	PN
A/Shenzhen/406H/06	Shenzhen	2.3.4	ABO36644	PN
A/chicken/Shanxi/2/2006	Shanxi	7	ABK34764	PN

[1] Hemagglutination inhibition (HI) assay was performed with viruses expressing native hemagglutinin (HA) and neuraminidase (NA).
[2] Pseudotype-based neutralization (PN) assay was performed with pseudotypes expressing codon-optimised HA from the indicated strain. All pseudotypes express the same N1NA derived from A/Thailand/1(KAN-1)/2004 virus.

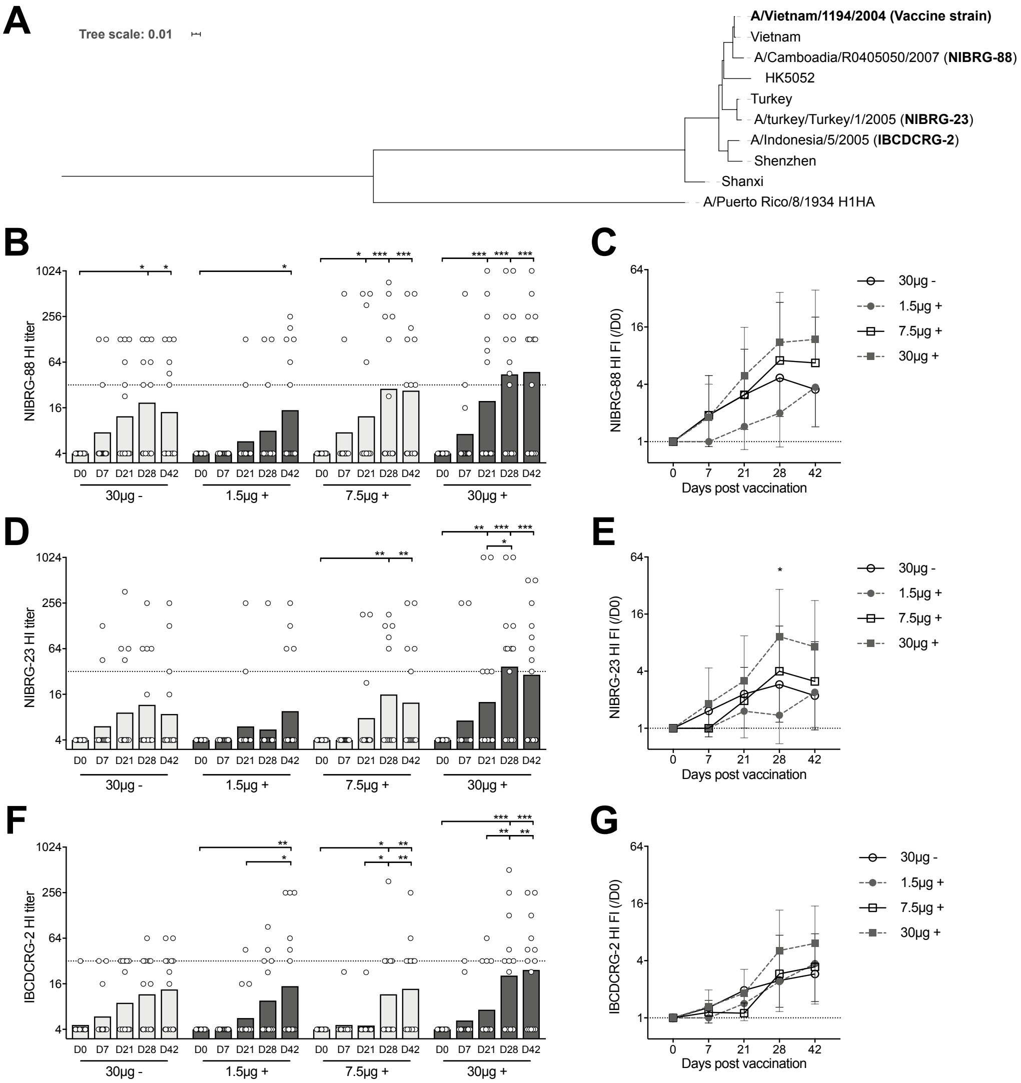

Figure S1 (related to Fig 1) Hemagglutination inhibiting antibodies to heterologous H5N1 viruses after vaccination.

(**A**) Phylogenetic tree showing the genetic divergence between H5HA from the vaccine strain, heterologous H5HAs tested in hemagglutination inhibition (HI) assay. The vaccine strain and heterologous H5N1 viruses tested in HI are highlighted in bold. (**B**-**G**) Cross-reactive hemagglutination inhibiting antibodies were measured against the heterologous H5N1 viruses NIBRG-88 (**B**-**C**), NIBRG-23 (**D**-**E**), and IBCDC-RG2 (**F**-**G**). HI titers (**B**, **D** and **F**) and fold-induction (FI) after vaccination (HI FI, /D0, **C**, **E**, and **G**) are shown. All antibody responses were measured using serum samples pre- (D0), 7 days (D7), 21 days (D21), 28 days (D28), and 42 days (D42) post-vaccination. The geometric mean titers are shown as bars, and each symbol represents one subject (**B**, **D** and **F**). The geometric means of fold-induction in each group ± geometric standard deviation as error bar are shown (**C**, **E**, and **G**). * P<0.05, ** P<0.01, *** P<0.001 (Antibody titers and fold-inductions were Ln transformed in statistical analyses. Turkey's multiple comparisons between pre-prime (D0) and post-prime (D7, D21, D28, D42), and between pre-boost (D21) and post-boost (D28 and D42) in each group were performed in two-way ANOVA in **B. D** and **F**. Turkey's multiple comparisons between 30 μg + and 1.5 μg + after prime (D7 and D21), and between 30 μg + and 30 μg - after boost (D28 and D42) were performed in two-way ANOVA in **C**, **E** and **G**). The horizontal dotted lines indicate HI titer of 32 (**B, D** and **F**), and fold-induction of 1 (**C**, **E** and **G**). Duplicates were performed in all experiments.

Supplementary table 2. Model summary of uni/multiple linear regression analyses for predicting mice protection by serologic immunity to influenza.

	Maximum body weight loss[1]				Lung viral load[2]			
Predictor(s)[3]	R	Adjusted R square	SE[4]	Sig.[5]	R	Adjusted R square	SE	Sig.
Uni-								
HI titer	0,807	0,644	4,835	**<0,001**	0,698	0,476	0,683	**<0,001**
HA stalk IgG	0,797	0,627	4,951	**<0,001**	0,629	0,383	0,742	**<0,001**
PN titer	0,917	0,837	3,276	**<0,001**	0,703	0,483	0,679	**<0,001**
NI titer	0,921	0,844	3,201	**<0,001**	0,768	0,581	0,611	**<0,001**
Multi-								
PN titer, HI titer, HA stalk IgG	0,917	0,829	3,35		0,742	0,52	0,654	
NI titer, HI titer, HA stalk IgG	0,923	0,841	3,229		0,769	0,564	0,623	
PN titer, NI titer	0,926	0,851	3,134		0,789	0,605	0,593	
PN titer, NI titer, HI titer, HA stalk IgG	0,926	0,845	3,195		0,813	0,63	0,574	
HI titer, HA stalk IgG	0,89	0,783	3,776		0,739	0,527	0,649	

[1] Maximum body weight loss (%) of 47 mice were used as dependent variable in regression analyses.
[2] Lung viral load ($TCID_{50}$/g, Ln transformed) of 48 mice were used as dependent variable in regression analyses.
[3] Serologic result(s) of group-and-time-point-wise pooled human sera were Ln transformed and centered before being used as independent variable(s) in regression analyses.
[4] Standard error of the estimate (SE).
[5] P value for significance. P<0,05 is highlighted in bold. Significance of each predictor in multiple linear regression is shown in the coefficient table (supplementary table 2).

Supplementary table 3. Coefficients of multiple linear regression analyses for predicting mice protection by serologic immunity to influenza.

	Maximum body weight loss[1]				Lung viral load[2]			
Predictors[3]	β[4]	t	Sig.[5]	VIF[6]	β	t	Sig.	VIF
PN titer, HI titer, HA stalk IgG								
PN titer	-0,948	-3,588	**0,001**	18,785	0,284	0,63	0,532	19,879
HI titer	0,021	0,125	0,901	7,488	-0,656	-2,347	**0,023**	7,642
HA stalk IgG	0,014	0,091	0,928	6,748	-0,465	-1,71	0,094	7,258
NI titer, HI titer, HA stalk IgG								
NI titer	-0,779	-4,144	**<0,001**	10,259	0,691	-2,195	**0,033**	10,662
HI titer	-0,042	-0,312	0,757	5,303	0,09	-0,401	0,69	5,413
HA stalk IgG	-0,126	-1,105	0,275	3,756	0,002	0,012	0,99	3,902
PN titer, NI titer								
PN titer	-0,401	-1,715	0,093	16,861	0,754	1,964	0,056	17,543
NI titer	-0,531	-2,27	**0,028**	16,861	-1,5	-3,908	**<0,001**	17,543
PN titer, NI titer, HI titer, HA stalk IgG								
PN titer	-0,456	-1,38	0,175	32,335	1,556	2,988	**0,005**	34,466
NI titer	-0,561	-2,298	**0,027**	17,659	-1,432	-3,755	**0,001**	18,487
HI titer	0,082	0,508	0,614	7,697	-0,504	-2,026	**0,049**	7,851
HA stalk IgG	0,013	0,086	0,932	6,749	-0,483	-2,022	**0,049**	7,261
HI titer, HA stalk IgG								
HI titer	-0,508	-5,778	**<0,001**	1,639	-0,5	-3,873	**<0,001**	1,654
HA stalk IgG	-0,48	-5,458	**<0,001**	1,639	-0,315	-2,439	**0,019**	1,654

[1] Maximum body weight loss (%) of 47 mice were used as dependent variable in regression analyses.
[2] Lung viral load ($TCID_{50}$/g, Ln transformed) of 48 mice were used as dependent variable in regression analyses.
[3] Serologic results of group-and-time-point-wise pooled human sera were Ln transformed and centered before being used as independent variables in multiple linear regression analyses.
[4] Standardized coefficients beta (β).
[5] P value for significance of each predictor. P<0,05 is highlighted in bold.
[6] The variance inflation factor (VIF) in collinearity statistics.

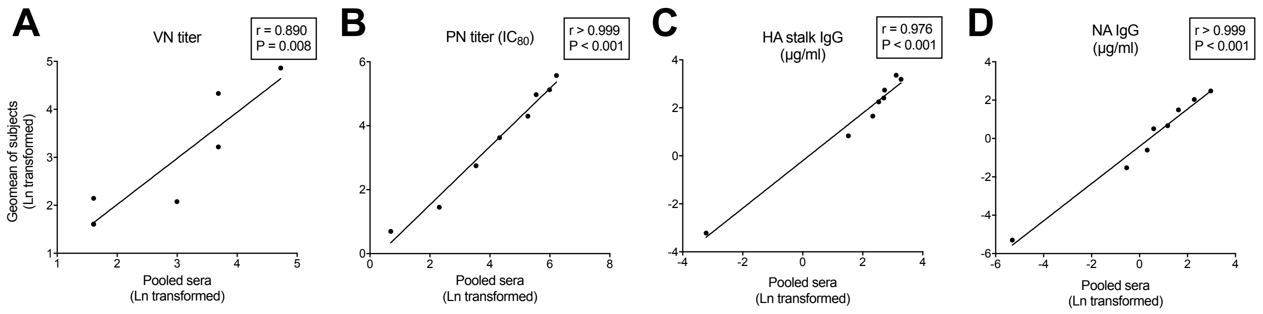

Figure S2 (related to Fig 5) Antibody parameters in the group-and-time-point-wise pooled human sera correlate with the antibody parameters pooled in silico. (**A**-**D**) The antibody parameters of group-and-time-point-wise pooled sera were measured directly in the pooled human sera. Antibody parameters pooled in silico were calculated as the geometric mean of antibody parameters from each individual subject in clinical trial put together the same way as sera were combined to make group-and-time-point-wise pooled human sera. Nonparametric Spearman correlation analyses were performed between the results from pooled human sera and the geometric mean of serum samples pooled in silico in virus neutralization assay against NIBRG-14 (**A**), pseudotype-based neutralization assay against Vietnam strain (**B**), HA stalk specific IgG concentration (**C**), and NA specific IgG concentration (**D**). Antibody results were Ln transformed in statistical analyses. Linear fitting curve is plotted as line; Spearman r and P values are noted in each correlation.
